# Real-world insights on a rare disease: adenoid cystic carcinoma of the breast

**DOI:** 10.3332/ecancer.2025.2010

**Published:** 2025-10-09

**Authors:** Cassio Murilo Hidalgo Filho, Mateus Marinho Nogueira Soares, Wesley Antonio Lopes de Lima, Laura Testa, Marcela Simonis Martins Ferrari, Renata Colombo Bonadio

**Affiliations:** 1 Instituto do Câncer do Estado de São Paulo, Universidade de São Paulo, Sao Paulo, SP 01246-000, Brazil; ‡ Contributed equally to the article; a https://orcid.org/0000-0002-7046-0059; b https://orcid.org/0009-0002-3890-5510; c https://orcid.org/0009-0009-3796-232X; d https://orcid.org/0000-0001-6080-7429; e https://orcid.org/0000-0002-3557-7921; f https://orcid.org/0000-0001-5818-922X

**Keywords:** breast cancer, adenoid cystic carcinoma breast, particular histology

## Abstract

**Background:**

Adenoid cystic carcinoma of the breast (ACCB) is a rare histological subtype of breast cancer characterised by unique clinical features and management challenges. ACCB remains poorly understood, with limited data on its epidemiology, treatment outcomes and prognostic factors. This study aimed to elucidate the landscape of ACCB in a real-world context.

**Methods:**

A retrospective cohort study was conducted on patients diagnosed with ACCB in a Brazilian cancer center between January 2007 and October 2021. Clinical and pathological data were systematically collected from electronic medical records. Statistical analyses were performed to identify factors associated with prognosis and assess the impact of treatment interventions.

**Results:**

Twenty-one female patients with confirmed ACCB were included in the study. The median age at diagnosis was 55.2 years. Most patients had basaloid (38.1%) or classic (19.0%) histological subtypes. Adjuvant radiotherapy was associated with a trend towards better recurrence-free survival among patients with localised disease HR 0.21, CI 95% 0.04–1.06, *p* = 0.059). In the metastatic setting, systemic chemotherapy used for breast cancer demonstrated limited efficacy, with a median progression-free survival of 1.8 to 2.8 months. Despite the overall poor prognosis, two patients with low-volume metastatic disease had long-term survival following local therapy.

**Conclusion:**

Given the rarity of ACCB and the absence of a standard management approach, this small study suggests a potential benefit of local therapies in adjuvant and metastatic settings while indicating the limited efficacy of systemic chemotherapy. Personalised treatment strategies tailored to ACCB are essential to optimising patient outcomes.

## Introduction

Adenoid cystic carcinoma of the breast (ACCB) stands as an exceptionally rare neoplasm, constituting a small fraction of primary breast cancers (less than 0.1%), being more common in women in the fifth and sixth decades of life [1]. Most of these ACCBs are localised diseases, demonstrating a unique histologic pattern with epithelial and myoepithelial components reminiscent of tumours originating in the salivary glands [2]. Despite being classified as a triple-negative breast cancer (TNBC) entity (lacking expression of estrogen receptor (ER), progesterone receptor (PR) and human epidermal growth factor receptor 2 (HER2)) and designated as a distinct basal-like tumour, ACCB is characterised by a low incidence of axillary involvement and distant metastasis [3].

Resistance to cytotoxic chemotherapy is a prominent characteristic observed in adenoid cystic carcinomas (ACC). Despite previous trials investigating the efficacy of tyrosine kinase inhibitors (TKIs) for this disease, there is currently no therapy specifically approved by the Food and Drug Administration for this histological subtype [4]. The scarcity of literature on ACCB has resulted in a notable gap in understanding the natural history of this malignancy. This highlights the need for comprehensive studies to elucidate its clinical features and address optimal management strategies, particularly given the absence of standardised treatment protocols.

The objective of this study was to delineate the epidemiological profile, treatment patterns and outcomes of patients diagnosed with ACCB. We provided a comprehensive description of the treatment management employed for these patients within the unique clinical setting of a tertiary cancer center in Latin America.

## Methods

### Study design and participants

A retrospective cohort study was conducted on patients diagnosed with ACCB who received treatment at the Instituto do Cancer do Estado de São Paulo (ICESP), Brazil, between January 2007 and October 2021. Inclusion criteria encompassed patients with a confirmed anatomopathological diagnosis of ACCB, which an experienced breast pathology group from ICESP reviewed. Patients who did not undergo surgical, radiotherapy (RT) and systemic therapy treatment were excluded. The study received approval from the local Institutional Review Board and Ethics Committee.

### Data collection

Patients were selected based on the International Classification of Diseases code C50 and underwent screening for adenoid cystic histology as indicated in the anatomopathological reports. Data was systematically recorded using a RedCap case report form. Extracted information from electronic medical records included demographic details (sex and age), cancer staging, Eastern Cooperative Oncology Group performance status, ACCB histologic subtype (basaloid, classical or other), histological grading, Ki-67, treatment received, intent of systemic therapy ((neo)adjuvant or palliative), number of treatment cycles, exposure to RT (both adjuvant and palliative settings), date of recurrence, date of progression, date of death and date of last follow-up.

### Outcomes and statistical analysis

Continuous variables were delineated by median and range. Categorical variables were presented using absolute numbers and proportions. For patients with early disease, recurrence-free survival (RFS) was calculated from the time of breast cancer disease until the date of local recurrence, distant recurrence or death, whichever occurred first. For those with advanced disease, overall survival (OS) was calculated from the time of diagnosis of metastatic or unresectable disease until the date of death from any cause. Patients without these events were censored at the date of the last follow-up. Survival probabilities were computed using the Kaplan–Meier method. The Cox regression model was used to evaluate prognostic factors. A significance threshold of *p* < 0.05 was adopted for statistical significance. The statistical analyses were performed using Stata Software, version 15.1 (StataCorp, Texas, USA, 2017).

## Results

### Patient's characteristics

A total of 21 patients with ACCB were identified. All patients had triple-negative tumours and were female. The cohort’s median age was 53.2 (range 38.8–78.6). Most (76.9%) declared themselves white. Approximately half (47.2%) had a history of breast or ovarian cancer among first and second-degree relatives. Germline testing was not available at the institution. Basaloid (38.1%) and classic (19.0%) were the most common histologic subtypes. Regarding HER2 status, only one patient (4.7%) was HER2-low, while 12 patients (57.1%) were HER2-zero and 8 patients (38.1%) were HER2-negative without detailed immunohistochemistry results. Table 1 describes the clinical and pathological characteristics of all included patients.

### Treatment patterns and outcomes in early-stage disease

Most patients (*n* =18–85,7%) included in the study had initial localised or locally advanced disease (stages I–III). Treatment patterns are detailed in Table 2. Four patients underwent neoadjuvant systemic chemotherapy, all with anthracycline and taxane-based regimens. Clinical response was identified in three patients (75%), while the other had stable disease. No progressive disease was observed during neoadjuvant therapy. Among these four patients, two underwent mastectomy and two underwent conservative surgery. All of them had residual disease in the surgical specimen. Among the 14 patients treated with upfront surgery, only three received adjuvant systemic therapy: two with an anthracycline-based regimen and one with cyclophosphamide, methotrexate and fluorouracil (CMF).

**Figure 1.**
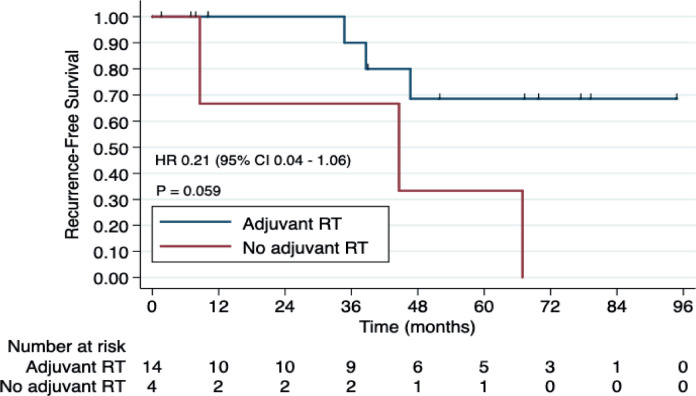
RFS according to adjuvant RT (yes versus no) among patients with ACCB. Abbreviations: RT, radiotherapy.

Fourteen patients (77.8%) received adjuvant RT, while the other four patients in the localised disease group were treated with mastectomy, and therefore, RT was not indicated. Adjuvant RT was associated with improved RFS (HR 0.21, CI 95% 0.04–1.06, *p* = 0.059). The 3-year RFS rates were 90.0% (95% CI 47.3%–98.5%) among patients who received adjuvant RT, in contrast to 66.6% (95% CI 5.4%–94.5%) observed among patients who did not undergo adjuvant RT (Figure 1).

Among the four patients who did not receive RT after mastectomy, three (75%) had an EFS event: one had a concomitant local and distant recurrence, one had a distant-only recurrence and the other died without recurrence. Considering the 14 patients who received adjuvant RT, three (21.4%) had an EFS event: one had a local followed by distant recurrence and the other two had distant-only recurrence.

ACCB subtype, tumour stage, histological grade, lymphovascular invasion, perineural invasion, Ki67 index and (neo)adjuvant chemotherapy were not associated with RFS (Table 3).

### Outcomes in metastatic disease

**Figure 2.**
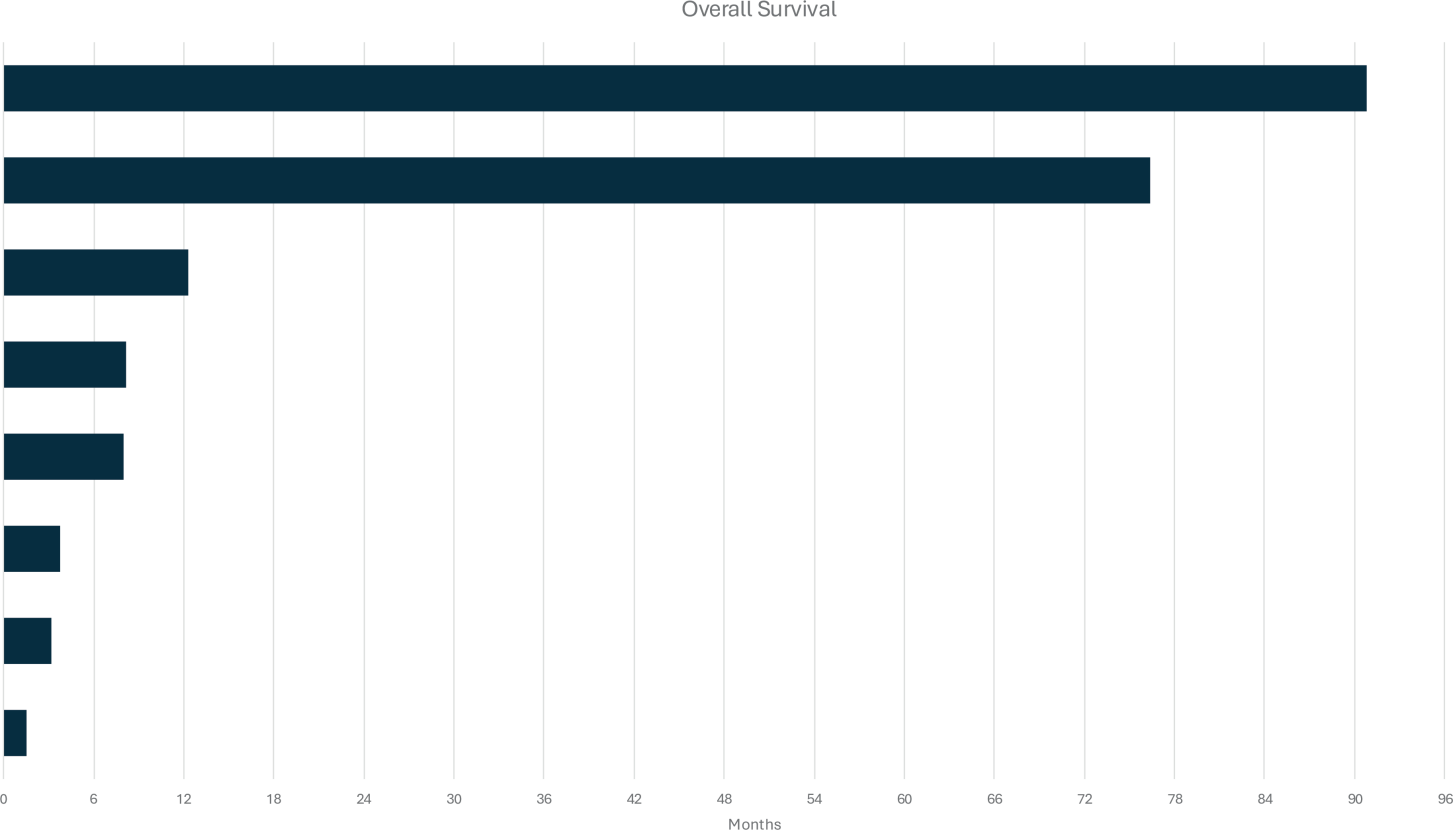
Swimmer's plot of the OS of patients with metastatic ACCB.

As described above, five patients (27,7%) in our cohort developed distant recurrence. The median OS from the time of metastasis diagnosis was 8.17 months, indicating a relatively poor prognosis for patients with metastatic ACCB (Figure 2).

Two patients with late recurrence received chemotherapy as first-line treatment with a short PFS and unfavourable outcomes, as detailed below. The first patient in this scenario was 46 years old, with a non-specified adenoid cystic subtype and had stage IIIb and histologic grade III at diagnosis. She underwent breast-conserving surgery followed by adjuvant doxorubicin cyclophosphamide and paclitaxel and developed distant recurrence at multiple sites (bone, liver and peritoneum) after 46 months. At the time of progression, she was treated with Cisplatin and Gemcitabine, yielding a PFS of 1.8 months.

The second patient was 39 years old with basaloid adenoid cystic subtype, had stage I at diagnosis and underwent mastectomy as the primary treatment – chemotherapy and RT were not indicated. The patient presented a late lung recurrence after a follow-up of 5 years from diagnosis (Supplementary Table 1), which was treated with local RT. She maintained a status of stable disease for 2 more years when she developed systemic progression; the treatment of choice was carboplatin plus paclitaxel in the first-line setting, yielding a PFS of 2.8 months. Both patients died from metastatic disease after 10 and 12 months from the start of first-line systemic therapy, respectively.

Two of the five patients who developed distant recurrences had exceptional survival outcomes. Both had low-volume metastatic disease and were treated with local therapies that controlled the disease for a long time.

One patient was 51 years old, with classic adenoid cystic subtype, stage II and histologic grading 1; she was treated with mastectomy, and no adjuvant chemotherapy or RT was indicated. After a follow-up of 44 months, she was diagnosed with local and pulmonary synchronous relapse. She was initially monitored, showing a slow growth rate of the lesions. Twenty-five months after the recurrence, surgical treatment for the relapses (local and lung) was performed. Later in follow-up, progressive lung disease was identified, and RT, consisting of 16 fractions of 250 cGy, was the treatment of choice. She received no systemic treatment. This patient died from metastatic disease after 135 months (11.2 years) from the initial diagnosis.

The second patient was 50 years old, with a non-specified adenoid cystic subtype, had stage II at diagnosis, histologic grading 2 and underwent local conservative breast surgery to which adjuvant RT was performed. After a follow-up of around 60 months, she developed local recurrence and was then treated with a total mastectomy. Later, in follow-up, she was diagnosed with a sternum recurrence, which was also treated with surgical resection. This patient died from metastatic disease after 175 months (14.6 years) from the initial diagnosis.

Three patients (14.2%) presented with metastatic disease at diagnosis. None was treated with chemotherapy. Two of them died after 3.8 and 8.1 months, while the third was lost to follow-up. The most frequent sites among all metastatic patients were bone (50%), liver (25%) and lung (25%). Contralateral breast, central nervous system, peritoneum and non-regional lymph node metastasis were found in one patient each (Supplementary Table 2).

## Discussion

In this study, we present a thorough analysis of the epidemiological profile of patients diagnosed with a rare subtype of breast cancer within a 15-year experience at a cancer center in Brazil. Most patients are diagnosed with early-stage disease, and a treatment approach comprising surgery followed by adjuvant RT was significantly associated with favourable long-term RFS outcomes.

The different subtypes of TNBC have heterogeneous molecular signatures, clinical characteristics and prognoses [5, 6]. Previous cohorts corroborate the low incidence of ACCB histological subtype compared to other special breast cancer histologies [7, 8]. Recent genomic studies have revealed that ACCB is frequently driven by chromosomal translocations involving the MYB gene, most notably the MYB–NFIB fusion. In some cases, MYBL1 rearrangements or MYB amplifications have also been described, contributing to aberrant MYB pathway activation. These alterations result in the overexpression of MYB/MYBL1 transcription factors, which regulate genes related to cell cycle progression, apoptosis inhibition and stem cell maintenance. Histologically, this activation is thought to underpin the cribriform or tubular architecture characteristic of ACCB. Although there are currently no approved therapies directly targeting the MYB pathway, its role as a diagnostic biomarker and future therapeutic target has gained increasing interest in translational research [9–11].

Understanding how ACCB behaves in different populations is crucial to contextualising our findings. When comparing our cohort to large population-based studies, important contrasts emerge. Our patients were younger (median age 53.2 years versus 62 years in SEER and 60 years in NCDB), and all tumours were triple-negative, whereas hormone receptor positivity was more prevalent in both SEER and NCDB [12, 13]. A comparative overview is provided in Supplementary Table 3.

The management of ACCB presents significant challenges due to its rarity and unique histological characteristics. Among patients with localised disease, only a minority received (neo)adjuvant chemotherapy, a decision typically based on individual clinical features and the discretion of the attending oncologist. However, the optimal role of systemic therapy in this context remains uncertain. Despite limited prospective data, retrospective studies have suggested a lack of significant benefit from systemic treatment [14]. Nevertheless, in selected cases, (neo)adjuvant chemotherapy may be considered based on pathological characteristics [15]. Our cohort reflects this variability, as three out of four patients who received systemic treatment demonstrated benefit in clinical/radiological response. Thus, while the utility of systemic therapy in early-stage ACCB warrants further investigation, our study suggests that some patients may derive clinical and radiological benefits from such interventions [16]. On the other hand, none of the patients had a complete pathologic response.

While the benefit of systemic therapy remains unclear, the role of local treatments, particularly RT, is better established. Our study revealed that patients who underwent adjuvant RT experienced better RFS. This finding aligns with previous research demonstrating improvements in survival outcomes among ACCB patients who received adjuvant RT [3, 17, 18]. Particularly, patients presenting high-risk features, such as T3-T4 tumours, high histological grade and older age (>60 years), seem to benefit from RT [19]. Notably, the rate of adjuvant RT in our cohort (77.8%) was higher than in the SEER (40.7%) and NCDB (47.1%) cohorts, which may reflect more standardised local protocols, differences in referral patterns or case selection at a high-complexity cancer center.

Despite its classification as a TNBC subtype, ACCB exhibits distinct clinical behaviour characterised by a relatively low incidence of distant metastasis in most reports [7]. In our study, however, the rate of distant recurrence was 27.7%, which is notably higher than in SEER (10.3%) and NCDB (12%) This discrepancy may be attributed to a higher prevalence of the basaloid subtype in our cohort—associated with more aggressive disease—although this histologic subclassification was not available in the SEER or NCDB datasets [12, 13]. Additionally, most of our patients were HER2 0, suggesting that HER2 expression does not contribute meaningfully to tumour progression in this subtype. Delays in presentation and diagnosis, which are common in public healthcare systems in low- and middle-income countries, may also have contributed to the higher recurrence burden observed in our cohort [15, 20, 21].

The short PFS observed in the two patients who underwent chemotherapy in the metastatic setting, 1.8 and 2.8 months, respectively, aligns with findings from previous studies showing the limited efficacy of cytotoxic therapy for ACC in general [22, 23]. Both patients received platinum-based combinations. Despite the prospective data regarding the activity of the TKI lenvatinib in ACC [4], none of the patients in our cohort received this therapy due to its unavailability in the Brazilian public health system.

For patients with advanced disease, local therapies such as surgical removal or RT remain valuable strategies, as demonstrated by two patients in our cohort, underscoring the need for alternative treatment approaches to improve outcomes in this setting [24]. These two patients in our cohort had an outstanding OS; both had oligometastatic disease and were treated with local RT with long disease control time.

One of our study strengths is that it is one of the few cohorts of ACCB patients from a lower-middle-income country and the largest reported in Latin America [25, 26]. Furthermore, we present a cohort with a high number of metastatic patients. The extended follow-up duration enabled a comprehensive analysis of patient outcomes over time, providing valuable insights into the disease course. However, it is essential to acknowledge the limitations inherent in our study. The retrospective design introduces inherent biases and limitations. The relatively small number of patients also challenges drawing definitive conclusions and generalising findings. Moving forward, multicentric collaborative prospective studies with larger sample sizes and detailed treatment protocols are warranted to validate our findings and provide more robust evidence to guide clinical practice effectively for this rare entity.

## Conclusion

In conclusion, our study provides valuable insights into the clinical characteristics and outcomes of patients with ACCD. Due to its rarity, ACCB poses significant challenges in management, necessitating further research to elucidate its natural history and optimise treatment strategies. Our results suggest that adjuvant RT may be a key component in reducing recurrence rates. Local therapy might also have a role in oligometastatic disease, while systemic chemotherapy demonstrates limited efficacy. Personalised treatment approaches tailored to the unique features of ACCB hold promise for improving patient outcomes in the future.

## Conflicts of interest

**Cassio Murilo Hidalgo Filho:** Speaker fees: AstraZeneca, Daiichi-Sankyo.

**Mateus Marinho Nogueira Soares, Wesley Antonio Lopes de Lima:** Declare no conflicts of interest.

**Marcela Simonis Martins Ferrari:** Financial support for educational programs from Roche.

**Laura Testa:** Speaker fees and honoraria for consulting or advisory functions: Daiichi-Sankyo, MSD, AstraZeneca, Pfizer, Lilly, Novartis. Financial support for educational programs and symposia: AstraZeneca, Roche, Gilead. Institutional Research grant: Novartis.

**Renata Colombo Bonadio:** Speaker fees and honoraria for consulting or advisory functions: Daiichi-Sankyo, Nestle Health Science, Addium, Gilead, MSD, BMS, AstraZeneca, Ache, Pfizer. Financial support for educational programs and symposia: AstraZeneca, Daiichi-Sankyo, MSD. Institutional Research grant: Novartis, AstraZeneca.

## Funding

No funding was received for this study.

## Author contributions

**Cassio Murilo Hidalgo Filho:** Investigation, methodology, draft writing, writing review and supervision. **Mateus Marinho Nogueira Soares, Wesley Antonio Lopes de Lima:** Investigation, data collection, draft writing, writing review and conceptualisation.** Marcela Simonis Martins Ferrari:** Conceptualisation, methodology, writing review and supervision. **Laura Testa:** Writing review and supervision. **Renata Colombo Bonadio:** Investigation, formal analysis, methodology, draft writing, writing review and conceptualisation.

## Figures and Tables

**Table 1.** Clinical characteristics of patients diagnosed with ACCB (*N* = 21).

Clinical feature	*N*	%
Age, Years – Median (range)	53.2 (38.8–78.6)	
Ki-67, % - Median (range)	25 (5–70)	
Body mass index – Median (range)	26.1 (18.9–41.6)	
Race Caucasian Black Brown	16 1 4	76.19 4.76 19.05
Previous pregnancy Yes No NA	12 1 8	57.14 4.76 38.10
Breast feeding history Yes No NA	6 3 12	28.57 14.29 57.14
Family history of breast or ovarian cancer No Yes – first degree relative Yes – second degree relative NA	10 5 4 3	47.61 23.80 19.04 14.28
Staging (8th AJCC) I II III IV	6 9 2 3	30.00 45.00 10.00 15.00
ACCB subtype Basaloid/solid Classic Other NA	8 4 2 7	38.10 19.05 9.52 33.33
HER2 status Score 0 Negative (Unavailable score) Low (Score 1)	12 8 1	57.14 38.10 4.76
Histological grading 1 2 3	6 12 3	28.57 57.14 14.29
Angiolymphatic invasion Yes No NA	1 17 3	4.76 80.95 14.29
Perineural invasion Yes No NA	5 13 3	23.81 61.90 3.00

Abbreviations: ACCB, Adenoid cystic carcinoma of the breast; NA, not available

**Table 2.** Treatment details of patients diagnosed with local or locally advanced ACCB treated in a tertiary cancer center in Brazil (*N* = 18).

Treatment	*N*	%
Neoadjuvant CT Yes No	4 14	22.22 77.78
ACCB resection Yes No	18 0	100.00 0.00
Type of primary surgery Mastectomy Conversative surgery	8 10	44.44 55.56
Type of axillary surgery Axillary dissection Sentinel node biopsy	4 14	22.22 77.78
Adjuvant CT Yes No	3 15	16.67 83.33
RT Yes No	14 4	77.78 22.22

ACCB: Adenoid cystic carcinoma of the breast, CT: chemotherapy

**Table 3.** Univariate analysis of prognostic factors for RFS among patients with early staging ACCB.

Variable	HR	95% CI	*p*
ACCB subtype (basaloid versus classic)	0.40	0.02–6.62	0.529
Tumour stage I II III	Ref 3.64 5.86	0.36–36.01 0.32–105.90	0.268 0.231
Histological grade 1 2 3	Ref 1.31 1.23	0.21–7.94 0.10–14.35	0.768 0.865
Lymphovascular invasion (yes versus no)	3.03	0.31–29.52	0.338
Perineural invasion (yes versus no)	2.37	0.39–14.40	0.347
Ki67 index (continuous)	1.02	0.97–1.06	0.309
Adjuvant RT (yes versus no)	0.21	0.04–1.06	0.059
(Neo) adjuvant CT (yes versus no)	2.47	0.44–13.66	0.298

ACCB: Adenoid cystic carcinoma of the breast, RT: radiotherapy, CT: chemotherapy

## Supplementary Information

**Supplementary Table 1.** Characteristic of patients with local or locally advanced adenoid cystic breast cancer - case series.

Patient nº	Staging	Grade	Subtype	Local treatment	(Neo)adjuvant treatment	Recurrence	Death
1	II	2	NA	Mastectomy	-	No	No
2	II	1	NA	Mastectomy	-	No	Yes
3	II	1	Classic	Mastectomy	-	Distance and local	Yes
4	I	2	Basaloid	Mastectomy	FAC	Distance	No
5	I	2	Classic	BCS	-	No	No
6	II	1	NA	BCS	-	No	No
7	II	1	Other	Mastectomy	AC-T	No	No
8	II	3	Basaloid	BCS	AC-T	No	No
9	I	2	Basaloid	BCS	-	No	No
10	I	2	Other	BCS	-	No	No
11	I	1	NA	BCS	-	No	No
12	III	2	Basaloid	Mastectomy	AC-T	No	No
13	II	2	Basaloid	BCS	-	No	No
14	II	3	Classic	Mastectomy	-	No	No
15	I	2	Classic	BCS	-	No	No
16	II	2	Basaloid	BCS	AC-T	Distance	No
17	III	3	NA	BCS	AC-T	Distance	No
18	II	2	NA	BCS*	CMF	Distance and new primary	Yes

* BCS was performed for the original tumour and the new primary

**Supplementary Table 2.** Characteristic of patients with de novo metastatic adenoid cystic breast cancer - case series.

Patient nº	Staging	Grade	Subtype	Systemic chemotherapy	Metastatic site	Death
**19**	**IV**	**2**	**Basaloid**	**No**	**Bone**	**Yes**
**20**	**IV**	**2**	**Basaloid**	**No**	**Contralateral breast**	**No**
**21**	**IV**	**2**	**Basaloid**	**No**	**Bone and lung**	**Yes**

**Supplementary Table 3.** Comparative characteristics of ACCB patients across cohorts.

Characteristic	Our cohort (*N* = 21)	SEER – Yang *et al* [27] (*N* = 1,036)	NCDB – Kulkarni *et al*. [13] (*N* = 933)
Median age (years)	53.2	62	60
Histological grade I–II	57.1%	46.7%	46%
HER2-negative	95.2%	98.2%	Not available
Hormone receptor-negative	100%	58.6% (ER); 65.3% (PR)	84.6% (ER−); 86.7% (PR−)
Basaloid subtype	38.1%	Not specified	Not specified
Stage I–II	85.7%	64.1%	Not available
Node-positive disease	0%	2.6%	5.1%
Adjuvant RT	77.8%	40.7%	47.1%
Adjuvant chemotherapy	16.6%	9.6%	11.3%
